# Hydrogen-Bonded Networks Based on Cobalt(II), Nickel(II), and Zinc(II) Complexes of N,N'-Diethylurea

**DOI:** 10.1155/2010/618202

**Published:** 2010-06-27

**Authors:** Labrini Drakopoulou, Catherine P. Raptopoulou, Aris Terzis, Giannis S. Papaefstathiou

**Affiliations:** ^1^Department of Chemistry, University of Patras, 265 04 Patras, Greece; ^2^Institute of Materials Science, National Centre of Scientific Research “Demokritos”, 153 10 Aghia Paraskevi Attikis, Greece; ^3^Laboratory of Inorganic Chemistry, Department of Chemistry, National and Kapodistrian University of Athens, Panepistimiopolis, 157 71 Zografou, Greece

## Abstract

N,N'-diethylurea (DEU) was employed as a ligand to form the octahedral complexes [M(DEU)_6_]^2+^ (M=Co, Ni and Zn). Compounds [Co(DEU)_6_](BF_4_)_2_ (**1**), [Co(DEU)_6_](CIO_4_)_2_ (**2**), [Ni(DEU)_6_](CIO_4_)_2_ (**3**), and [Zn(DMU)_6_](CIO_4_)_2_ (**4**) have been prepared from the reactions of DEU and the appropriate hydrated metal(II) salts in EtOH in the presence of 2,2-dimethoxypropane. Crystal structure determinations demonstrate the existence of [M(DEU)_6_]^2+^ cations and CIO_4_^−^ (in **2–4**) or BF_4_^−^ (in **1**) counterions. The [M(DEU)_6_]^2+^ cations in the solid state are stabilized by a *pseudochelate* effect due to the existence of six strong intracationic N-H ⋯ O_(DEU)_ hydrogen bonds. The [M(DEU)_6_]^2+^ cations and counterions self-assemble to form hydrogen-bonded 2D architectures in **2–4** that conform to the **kgd** (kagome dual) network, and a 3D hydrogen-bonded **rtl** (rutile) network in **1**. The nature of the resulting supramolecular structures is influenced by the nature of the counter-ion. The complexes were also characterized by vibrational spectroscopy (IR).

## 1. Introduction

**Scheme 1:**
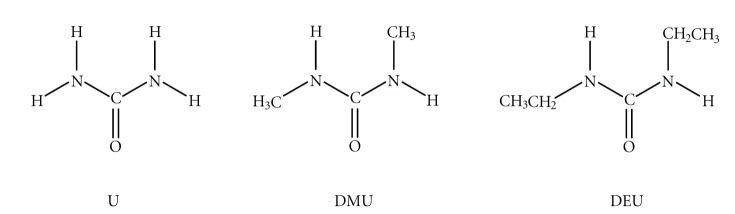
Ligands discussed in the text (U = urea, DMU = N,N'-dimethylurea and DEU = N,N'-diethylurea).

In 1828, Wöhler attempted to synthesize ammonium cyanate by reacting silver isocyanate (AgNCO) with ammonium chloride (NH_4_Cl). The outcome of this failed attempt was urea H_2_NCONH_2_ (U, Scheme 1) which represents the first organic molecule synthesized in the laboratory from purely inorganic materials [1]. Urea has also been recognized as the first organic molecule that was synthesized without the involvement of any living system [1]. Nowadays, urea represents not only an important molecule in biology [2] but also an important raw material in chemical industry [3].

**Scheme 2:**
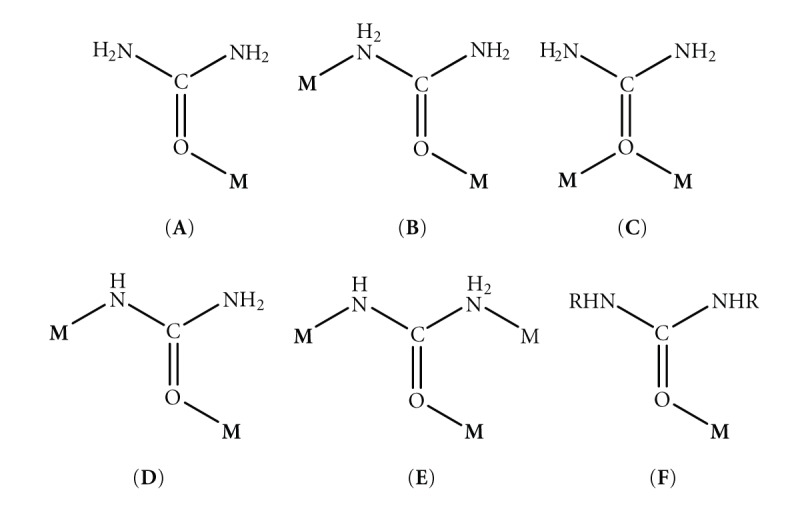
The crystallographically established coordination modes of urea (U) and its symmetrically substituted alkyl derivatives (RHNCONHR).

Restricting further discussion to the coordination chemistry of urea and its substituted derivatives, metal-urea complexes have attracted considerable interest since the discovery of the active site of urease, a metalloenzyme that catalyzes the hydrolysis of urea into carbon dioxide and ammonia [4, 5]. Considerable efforts have been devoted to devise useful bioinorganic models for the active site of urease and provide information for the intermediates and its catalytic mechanism. That in turn drove to the structural and spectroscopic characterization of many metal-urea complexes [6]. Urea usually coordinates as a monodentate ligand through the oxygen atom, forming a C=O⋯M angle considerably smaller than 180°, in accordance with the *s* *p* ^2^ hybridization of the O atom (**A** in Scheme 2). The rare N,O-bidentate coordination mode (**B** in Scheme 2) has been found in a very limited number of cases [7, 8], while in [Hg_2_Cl_4_U_2_] each U molecule bridges the two Hg^II^ atoms through the oxygen atom [9] (**C** in Scheme 2). Of particular chemical/biological interest is the ability of U to undergo metal-promoted deprotonation [4, 10]; the monoanionic ligand H_2_NCONH^−^ adopts the *μ*_2_ (**D** in Scheme 2) and *μ*_3_ (**E** in Scheme 2) coordination modes. The N,N'-alkyl symmetrically substituted derivatives of urea (RHNCONHR), such as the N,N'-dimethylurea (DMU) and N,N'-diethylurea (DEU) (Scheme 1) have only been found to coordinate as monodentate ligands through the oxygen atom (**F** in Scheme 2).

Urea and its substituted derivatives have been extensively studied within the frame of organic crystal engineering due to their ability to form extended hydrogen bonded frameworks. In particular, symmetrically substituted ureas (i.e., RHNCONHR) form *α*-networks with each urea molecule donating two hydrogen bonds and “chelating” the carbonyl oxygen of the next molecule in the network. In contrast to the great number of studies concerning free ureas [11–15], little is known about the supramolecular structures based on hydrogen bonding interactions between simple metal-ureas complexes. Over the last decade, we have been studying the coordination chemistry of urea and its symmetrically substituted derivative DMU [16–21]. In all cases, ureas form stable complexes which are further connected to create extended frameworks by intermolecular/interionic hydrogen bond interactions. Despite the large number of metal-urea complexes which have been structurally characterized, the metal-DMU complexes are considerably less studied while there only three reports with crystal structures of metal-DEU complexes [22–24]. In this report we present our first results from the study of metal-DEU complexes, extending the known crystal structures of metal-DEU complexes to seven.

## 2. Experiments

All manipulations were performed under aerobic conditions using materials and solvents as received. IR spectra were recorded on a Perkin-Elmer PC16 FT-IR spectrometer with samples prepared as KBr pellets. C, H and N elemental analyses were performed with a Carlo Erba EA 108 analyzer.

**Caution**

*Perchlorate salts are potentially explosive. Although no detonation tendencies have been observed in our experiments, caution is advised and handling of only small quantities is recommended.*

**[Co(DEU)_6_](BF_4_)_2_ (1)**

A pink solution of Co(BF_4_)_2_ *·*6H_2_O (0.68 g, 2.0 mmol) in EtOH (30 mL) and dimethoxypropane (DMP) (2.5 mL) was refluxed for 20 minutes, cooled to room temperature and then treated with solid DEU (1.40 g, 12 mmol). No noticeable colour change occurred. The reaction mixture was refluxed for a further 15 minutes, cooled to room temperature, and layered with Et_2_O (30 mL). Slow mixing gave pink crystals suitable for X-ray crystallography, which were collected by filtration, washed with cold EtOH (2 mL) and Et_2_O, and dried *in vacuo *over CaCl_2_. Typical yields were in the 70–80% range. Found %: C, 38.96; H, 7.59; N, 17.90. Calc % for C_30_H_72_N_12_O_6_CoB_2_F_8_: C, 38.77; H, 7.81; N, 18.08. IR data (KBr, cm^−1^): 3332 sb, 2976 s, 2934 m, 2878 m, 1626 vs, 1576 vs, 1482 w, 1454 m, 1380 m, 1338 m, 1294 m, 1160 m, 1142 m, 1110 sb, 1032 s, 922 w, 890 w, 768 m, 578 mb.

**[Co(DEU)_6_](ClO_4_)_2_ (2)**

A pink-red solution of Co(ClO_4_)_2_ *·*6H_2_O (0.73 g, 2.0 mmol) in EtOH (20 mL) and dimethoxypropane (DMP) (2.5 mL) was refluxed for 20 minutes, cooled to room temperature and then treated with solid DEU (1.40 g, 12 mmol). No noticeable colour change occurred. The reaction mixture was refluxed for a further 20 minutes, cooled to room temperature, and layered with Et_2_O (50 mL). Slow mixing gave pink crystals suitable for X-ray crystallography, which were collected by filtration, washed with cold EtOH (2 mL) and Et_2_O, and dried *in vacuo *over CaCl_2_. Typical yields were in the 75–85% range. Found %: C, 37.92; H, 7.49; N, 17.80. Calc % for C_30_H_72_N_12_O_14_CoCl_2_: C, 37.74; H, 7.60; N, 17.60. IR data (KBr, cm^−1^): 3332 sb, 2972 s, 2934 m, 2876 w, 1628 vs, 1570 vs, 1482 w, 1452 w, 1378 w, 1338 w, 1296 w, 1264 w, 1142 s, 1114 s, 1086 s, 922 w, 890 w, 768 w, 626 m.

**[Ni(DEU)_6_](ClO_4_)_2_ (3)**

A pale green solution of Ni(ClO_4_)_2_ *·*6H_2_O (0.73 g, 2.0 mmol) in EtOH (15 mL) and dimethoxypropane (DMP) (2.5 mL) was refluxed for 15 minutes, cooled to room temperature and then treated with solid DEU (1.40 g, 12 mmol). No noticeable colour change occurred. The reaction mixture was refluxed for a further 20 minutes, cooled to room temperature, and layered with Et_2_O (30 mL). Slow mixing gave green crystals suitable for X-ray crystallography, which were collected by filtration, washed with cold EtOH (2 mL) and Et_2_O, and dried *in vacuo *over CaCl_2_. Typical yields were in the 75–85% range. Found %: C, 37.90; H, 7.45; N, 17.82. Calc % for C_30_H_72_N_12_O_14_NiCl_2_: C, 37.75; H, 7.60; N, 17.61. IR data (KBr, cm^−1^): 3328 sb, 2976 m, 2934 w, 2876 w, 1636 vs, 1570 vs, 1508 w, 1450 m, 1380 w, 1334 w, 1268 m, 1146 s, 1118 s, 1086 s, 922 w, 772 w, 626 m.

**[Zn(DEU)_6_](ClO_4_)_2_ (4)**

A colourless solution of Zn(ClO_4_)_2_ *·*6H_2_O (0.74 g, 2.0 mmol) in EtOH (10 mL) and dimethoxypropane (DMP) (2.5 mL) was refluxed for 20 minutes, cooled to room temperature, and then treated with solid DEU (1.40 g, 12 mmol). The colourless reaction mixture was refluxed for a further 20 minutes, cooled to room temperature, and layered with Et_2_O (25 mL). Slow mixing gave colourless crystals suitable for X-ray crystallography, which were collected by filtration, washed with cold EtOH (2 mL) and Et_2_O, and dried *in vacuo *over CaCl_2_. Typical yields were in the 75–85% range. Found %: C, 37.62; H, 7.39; N, 17.60. Calc % for C_30_H_72_N_12_O_14_ZnCl_2_: C, 37.49; H, 7.55; N, 17.49. IR data (KBr, cm^−1^): 3340 sb, 2972 s, 2932 m, 2876 w, 1624 vs, 1582 vs, 1484 w, 1456 w, 1380 w, 1334 w, 1262 m, 1144 s, 1114 s, 1088 s, 924 w, 772 w, 636 m.

### 2.1. X-ray Crystallography

 X-ray data were collected at 298 K using a Crystal Logic Dual Goniometer diffractometer with graphite-monochromated Mo-*K_a_* radiation (*λ* *= *0.71073 Å). Lorentz, polarization, and *Ψ*-scan absorption corrections were applied using Crystal Logic software. The structures were solved by direct methods using SHELXS-86 [25] and refined by full-matrix least-squares techniques on *F* ^2^ with SHELXL-97 [26]. Details of the data collection and refinement are given in Table 1. Topological analysis of the nets was performed using TOPOS program package [27, 28].

## 3. Results and Discussion

### 3.1. Synthetic Comments

The preparation of the three complexes reported here is summarized in (1):

(1) $${\text{MX}}_{2}\cdot 6{\text{H}}_{2}\text{O}+6\text{DEU}\underset{\text{T},\;\;\text{DMP}}{\overset{\text{EtOH}}{\to }}\left[\text{M}{\left(\text{DEU}\right)}_{6}\right]{\text{X}}_{2}+6{\text{H}}_{2}\text{O}$$

M = Co, X = BF_4_ (**1**); M = Co, X = ClO_4_ (**2**); M = Ni, X = ClO_4_ (**3**); M = Zn, X = ClO_4_ (**4**).

2,2-dimethoxypropane (DMP), is a known dehydrating agent which under heating eliminates the possibility of [M(H_2_O)_6_]^2+^ formation in solution.

Complexes **1**–**4 **seem to be the only products from the MX_2_ *·*6H_2_O/DEU reaction systems (M=Co, Ni, Zn, X= ClO_4_ and M=Co, X=BF_4_). Changing the solvent from EtOH to MeCN to THF and Me_2_CO as well as the DEU : M^II^ reaction ratio from 6 : 1 to 12 : 1, 8 : 1, 4 : 1 and 3 : 1 does not seem to influence the identity of the products.

### 3.2. Description of Structures

**Figure 1:**
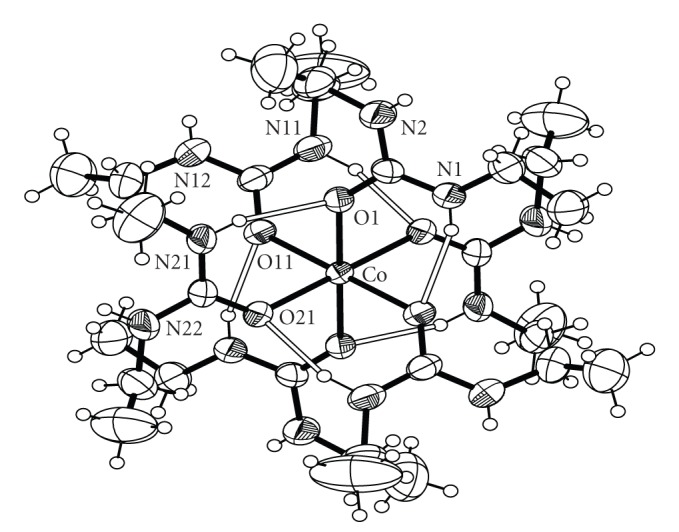
An ORTEP representation of the cation [Co(DEU)_6_]^2+^ present in complex **1**. Open bonds indicate intramolecular hydrogen bonds. The symmetry-equivalent atoms are not labeled.

**Figure 2:**
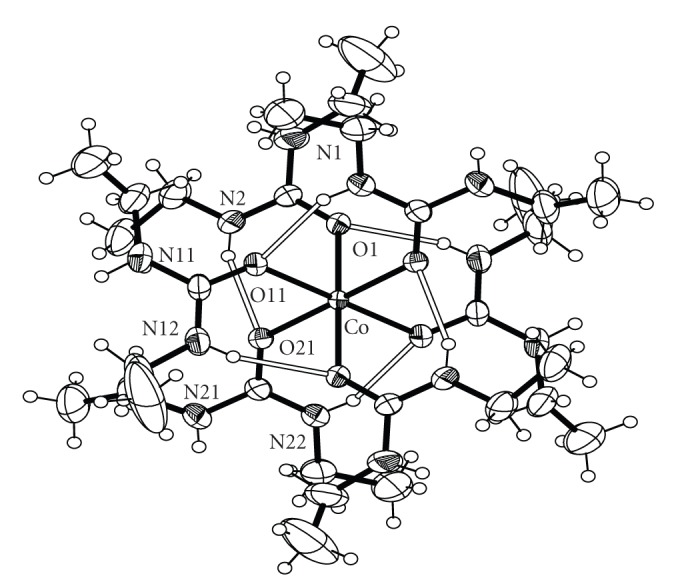
An ORTEP representation of the cation [Co(DEU)_6_]^2+^ present in complex **2**. Open bonds indicate intramolecular hydrogen bonds. The symmetry-equivalent atoms are not labeled.

**Figure 3:**
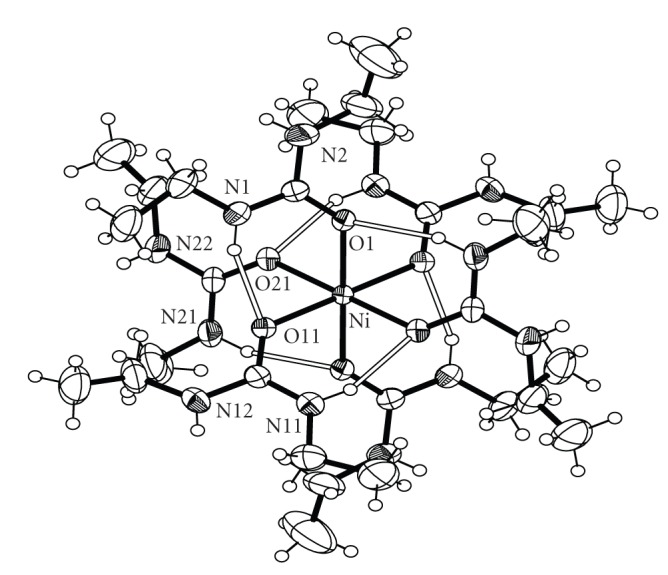
An ORTEP representation of the cation [Ni(DEU)_6_]^2+^ present in complex **3**. Open bonds indicate intramolecular hydrogen bonds. The symmetry-equivalent atoms are not labeled.

**Figure 4:**
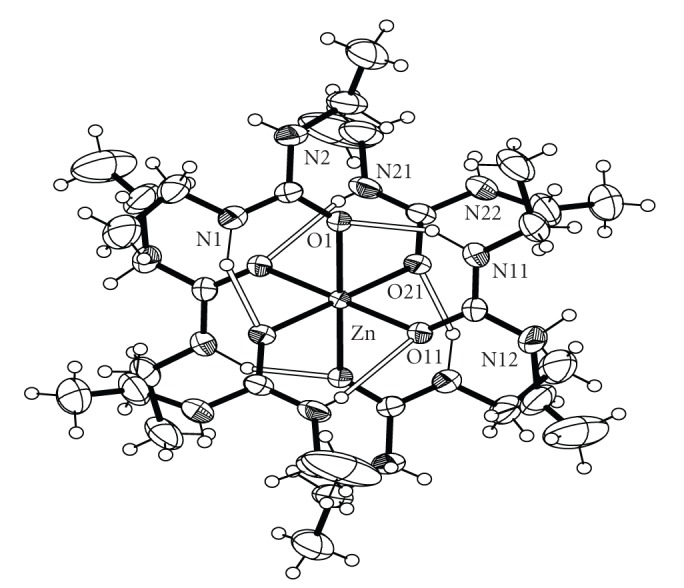
An ORTEP representation of the cation [Co(DEU)_6_]^2+^ present in complex **4**. Open bonds indicate intramolecular hydrogen bonds. The symmetry-equivalent atoms are not labeled.

Bond distances and angles for complexes **1**, **2**, **3** and **4** are listed in Tables 2, 3, 4, and 5, respectively. ORTEP plots of the cations [Co(DEU)_6_]^2+^, [Ni(DEU)_6_]^2+^, and [Zn(DEU)_6_]^2+^ present in complexes **1**, **2**, **3**, and **4** are shown in Figures 1, 2, 3, and 4, respectively. Details of the hydrogen bonds of **1**, **2**, **3**, and **4** are provided in Tables 6, 7, 8, and 9, respectively. Complexes **2**, **3,** and **4** crystallise in the triclinic space group *P* $\overset{®}{1}$ and are isostructural. Complex **1** crystallizes in the monoclinic space group *P*2_1_/c. The structures of **2**–**4** consist of almost perfect octahedral [M(DEU)_6_]^2+^ cations and ClO_4_^−^ counterions, while the same [M(DEU)_6_]^2+^ cation and BF_4_^−^ anions are present in the structure of **1**. In all four structures, the metal ion sits on an inversion centre and is surrounded by six O-bonded DEU ligands. The M–O_(DEU)_ bond distances in **1**–**4** are comparable to those in [M(DMU)_6_]^2+^ [17, 18]. The average M–O_(DEU)_ bond lengths change according to the sequence **1** [2.098 Å] *≅* **2** [2.096 Å] >**3** [2.072 Å] <**4** [2.108 Å] following the Irving-Williams series [29]. The DEU molecules in **1**–**4** are coordinated in a bent fashion forming C=O⋯M angles ranging from 127.6° to 132.5°. This is the usual way of coordination of urea and its derivatives and has been observed in the similar [M(DMU)_6_]X_2_ complexes [16–21]. Linearly or approximately linearly coordinated ureas are rare and have been observed only in a few cases [21]. There are six strong intramolecular (intracationic) hydrogen bonds inside each cation with atoms N(1), N(11), and N(21) (and their symmetry equivalents) as donors, and atoms O(1), O(11) and O(21) (and their symmetry equivalents) as acceptors for **1**, **3** and **4** and N(2), N(12) and N(22) (and their symmetry equivalents) as donors, and atoms O(1), O(11) and O(21) (and their symmetry equivalents) as acceptors for **2**. These intracationic hydrogen bonds create six-membered *pseudo*chelate rings providing extra stabilization to the [M(DEU)_6_]^2+^ cation. Overall the structural characteristics, that is, bond distances, agnles and intracation hydrogen bonding interactions in the [M(DEU)_6_]^2+^ resemble those found in the [M(DMU)_6_]^2+^ cations [17, 18] with an exception regarding two additional C-H ⋯ O H-bonds (and their symmetry equivalent) found in [Co(DMU)_6_](ClO_4_)_2_ and [Co(DMU)_6_](BF_4_)_2_ [18]. Complexes **1**–**4** extend to seven the number of structurally characterised DEU compounds. The three, previously structurally characterised, compounds are [SnBr_4_(DEU)_2_] [22], [Fe(DEU)_6_](ClO_4_)_2_ [23] and [Mn(DEU)_6_][MnBr_4_] [24]. Complexes **2**–**4** are isostructural to [Fe(DEU)_6_](ClO_4_)_2_. The average Fe-O_DEU_ bond distance is 2.105 following the Irving–Williams series as stated above.

**Figure 5:**
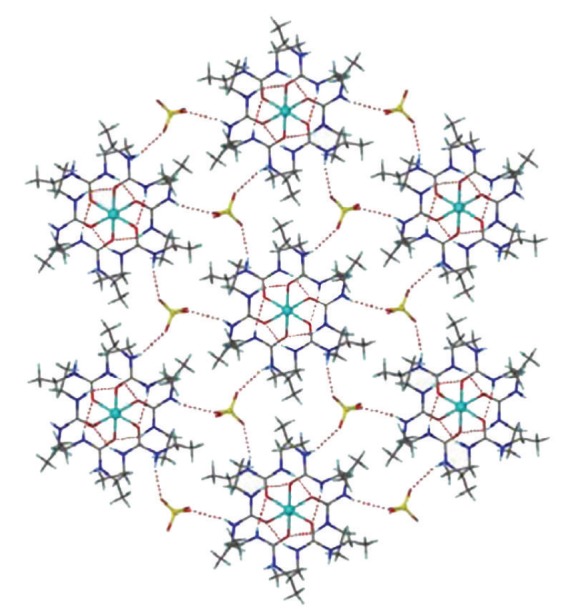
A view of the 2D framework formed by hydrogen bonding between the [Zn(DEU)_6_]^2+^ cations and the ClO_4_^−^ anions in **4**. The same framework is adopted by complexes **2** and **3**.

**Figure 6:**
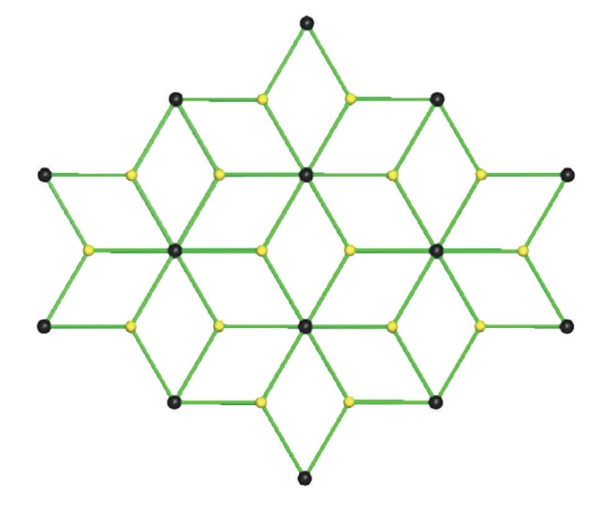
A view of the 2D hydrogen-bonded **kgd**-(4^3^)_2_(4^6^.6^6^.8^3^) net adopted by complexes **2**–**4**. Black spheres represent the 6-connected [M(DEU)_6_]^2+^ cations [M = Co (**2**), Ni (**3**) and Zn (**4**)] and yellow spheres the 3-connected ClO_4_^−^ anions.

Although the intracationic H-bonding interactions are the same along the [M(DEU)_6_]^2+^ series as well as very similar with those found in the [M(DMU)_6_]^2+^ cations, the intermolecular/interionic interactions are quite different. That the complexes **2**–**4** are isostructural implies that the interionic hydrogen bonding interactions are the same. Therefore, only the hydrogen bonding of the representative complex **4** will be discussed. The [Zn(DEU)_6_]^2+^ and ClO_4_^−^ ions in **4 **have assembled to create an infinite 2D network through three crystallographically independent intermolecular (interionic) N-H⋯O_(perchlorate)_ hydrogen bonds (and their symmetry related) (Figure 5). Each perchlorate accepts three hydrogen bonds with the O(31), O(32), and O(33) atoms acting as hydrogen bond acceptors while each [Zn(DEU)_6_]^2+^ connects to six ClO_4_^−^ anions through the remaining N-H groups (Figure 5). As a consequence of the participation of O(31), O(32), and O(33) in hydrogen bonding, the Cl-O(31), Cl-O(32), and Cl-O(33) bond lengths [1.402(1), 1.318(1) and 1.440(1) Å, resp.] are slightly longer than the Cl-O(34) [1.290(1) Å]. In this arrangement, a binodal (3,6)-connected network forms with Schläfli symbol (4^3^)_2_(4^6^.6^6^.8^3^) (Figure 6). This two-dimensional (2D) hydrogen-bonded **kgd** net is the dual of the kagome **kgm**-(3.6.3.6) net. It is worth noting that the 2D network adopted by **2**–**4** was not adopted by any of the [M(DMU)_6_](ClO_4_)_2_ complexes [17, 18] suggesting that the substitution of DMU by DEU substantially changes the intermolecular (interionic) interactions probably due to the larger ethyl groups (in DEU) instead of the smaller methyl groups (in DMU). Similar 2D networks have been adopted by [Zn(DMU)_6_](ClO_4_)_2_ [17] and [Co(DMU)_6_](BF_4_)_2_ [18] with the ClO_4_^−^ and the BF_4_^−^ anions acting as 3-connected nodes and the [M(DMU)_6_]^2+^ acting as 6-connected nodes but the connections are achieved through two N-H⋯X and one C-H⋯X hydrogen bonds (and their symmetry equivalents), (X = O_(perchlorate)_ or F_(tetrafluoroborate)_, resp.).

**Figure 7:**
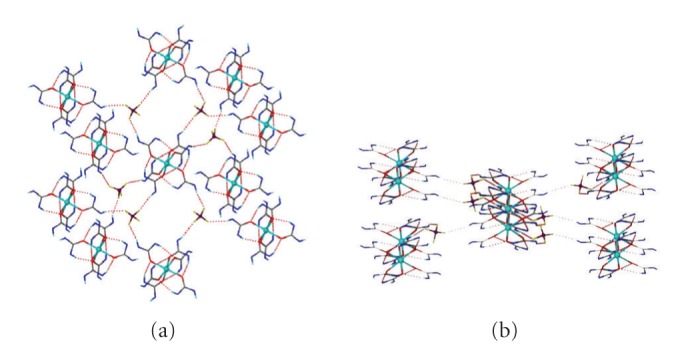
Views of the 3D framework formed by hydrogen bonds between the [Co(DEU)_6_]^2+^ cations and the BF_4_^−^ anions in **1**.

**Figure 8:**
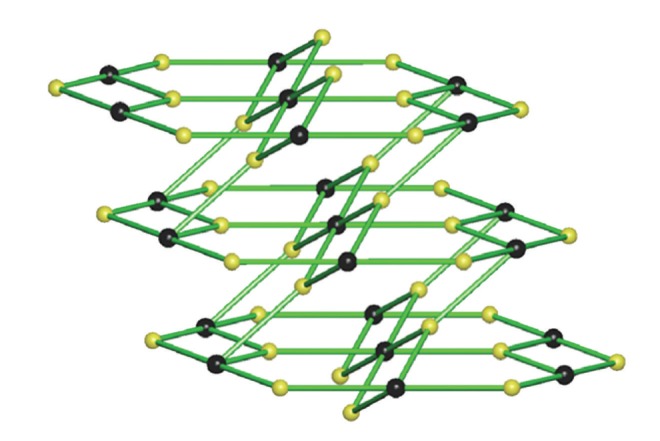
A view of the binodal 3D hydrogen-bonded **rtl**-(4.6^2^)_2_(4^2^.6^10^.8^3^) net that **1 **adopts. Black spheres represent the 6-connected [Co(DEU)_6_]^2+^ cations and yellow spheres the 3-connected BF_4_^−^ anions.

The intermolecular hydrogen bonding interactions in **1** are far more interesting that those in **2**–**4**. The [Co(DEU)_6_]^2+^ and the BF_4_^−^ anions have assembled to create a three-dimensional (3D) hydrogen-bonded framework through three crystallographically independent intermolecular (interionic) N-H⋯F_(tetrafluoroborate)_ hydrogen bonds (and their symmetry equivalents). Each BF_4_^−^ accepts three hydrogen bonds with the F(1), F(2) and F(3) atoms acting as hydrogen bond acceptors while each [Co(DEU)_6_]^2+^ connects to six BF_4_^−^ anions through the remaining N-H groups (Figure 7). In this arrangement, a (3,6)-connected network forms with the [Co(DEU)_6_]^2+^ cations acting as the 6-connected nodes and the BF_4_^−^ anions as the 3-connected nodes. Although the connectivity of each ion seems identical to that found in **2**–**4**, the arrangement of the [Co(DEU)_6_]^2+^ and BF_4_^−^ ions is quite different resulting in a binodal 3D hydrogen-bonded network with a rutile (**rtl**) topology [30, 31] and Schläfli symbol (4.6^2^)_2_(4^2^.6^10^.8^3^) (Figure 8). It is worth noting that none of the [M(DMU)_6_]X_2_ complexes [17, 18] adopts a 3D net.

### 3.3. Vibrational Spectra of the Complexes

Table 10 gives diagnostic IR bands of the free ligand and complexes **1**–**4**. Assignments have been given in comparison with the data obtained for the free DMU [32], the free DEU [33] and its Co(II) and Ni(II) complexes [34]. The bands with **ν**(CN) character are situated at higher wavenumbers in the spectra of **1**–**4** than for free DEU, whereas the **ν**(CO) band shows a frequency decrease. These shifts are consistent with oxygen coordination, suggesting the presence of ^+^N=C-O^−^ resonant forms [17, 18]. Upon coordination *via* oxygen, the positively charged metal ion stabilizes the negative charge on the oxygen atom; the NCO group now occurs in its polar resonance form and the double bond character of the CN bond increases, while the double bond character of the CO bond decreases, resulting in an increase of the CN stretching frequency with a simultaneous decrease in the CO stretching frequency [17, 18]. The *ν*_3_(F_2_) [*ν*_*d*_(BF)] and *ν*_4_(F_2_) [*δ*_d_(FBF)] vibrations of the tetrahedral (point group T_d_) BF_4_^−^ anion appear at 1100-1000 and at 522–580 cm^−1^ (broad bands), respectively, in the IR spectrum of **1 **[35]. The IR spectra of **2**–**4 **exhibit strong bands at ~1100 and 626 cm^−1^ due to the *ν*_3_(F_2_) and *ν*_4_(F_2_) vibrations, respectively, of the uncoordinated ClO_4_^−^ [35]. The broad character and splitting of the band at ~1100 cm^−1^ indicate the involvement of the ClO_4_^−^ ion in hydrogen bonding as it was established crystallographically (see above).

## 4. Conclusions

Following our studies on the coordination chemistry of urea (U) and N,N'-dimethylurea (DMU), N,N'-diethylurea (DEU) was employed as a ligand to form the stable octahedral complexes [M(DEU)_6_]^2+^ with cobalt(II), nickel(II) and zinc(II). The structural characteristics of the [M(DEU)_6_]^2+^ cation are very similar to the DMU analogs, that is, [M(DMU)_6_]^2+^. All six DEU molecules are coordinated to metal centre in a bent fashion forming a C=O⋯M angle of ~130°, while six strong intracationic N-H ⋯ O_(DEU)_ hydrogen bonds stabilize the [M(DEU)_6_]^2+^ cations by creating six six-membered *pseudo*chelate rings. The [M(DEU)_6_]^2+^ cations and counterions (ClO_4_^−^ or BF_4_^−^) self-assemble to form extended hydrogen-bonded architectures *via *3 unique N-H⋯X hydrogen bonds, (X = O_(perchlorate)_ or F_(tetrafluoroborate)_). The nature of the resulting supramolecular architectures is influenced by the nature of the counter-ion since the presence of ClO_4_^−^ counter-ions gives rise to the formation of 2D hydrogen-bonded networks that conform to the **kgd** net while the presence of BF_4_^−^ counter-ions results in a 3D hydrogen-bonded net with an **rtl** topology. By comparing the supramolecular architectures of the [M(DEU)_6_]X_2_ (X=ClO_4_ or BF_4_) and the [M(DMU)_6_]X_2_ (X=ClO_4_ or BF_4_) we can conclude that the substitution of DMU by DEU considerably affected the nature of the hydrogen-bonded networks. We are presently pursuing our studies on the coordination chemistry of urea and its symmetrically or unsymmetrically substituted alkyl derivatives to generate a rich variety of hydrogen-bonded networks.

## Figures and Tables

**Table 1:** Crystal data and structure refinement for **1**–**4**.

Compound	**1**	**2**	**3**	**4**
Empirical formula	C_30_H_72_B_2_CoF_8_N_12_O_6_	C_30_H_72_CoCl_2_N_12_O_14_	C_30_H_72_NiCl_2_N_12_O_14_	C_30_H_72_ZnCl_2_N_12_O_14_
Formula weight	929.52	954.81	954.59	961.26
Crystal size	0.10 × 0.20 × 0.20	0.10 × 0.15 × 0.20	0.10 × 0.15 × 0.20	0.10 × 0.20 × 0.20
Crystal system	monoclinic	triclinic	triclinic	triclinic
Space group	*P*2_1_/c	*P* $\;\overset{®}{1}$	*P* $\;\overset{®}{1}$	*P* $\;\overset{®}{1}$
**θ** range for data	1.85 ≤ *θ* ≤ 25.00	1.93 ≤ **θ** ≤ 25.00	1.76 ≤ **θ** ≤ 25.00	1.76 ≤ **θ** ≤ 25.00
collection. °				
*a*, Å	9.495(3)	13.341(5)	9.063(3)	9.092(6)
*b*, Å	22.063(7)	11.935(4)	11.951(6)	11.978(9)
*c*, Å	12.615(4)	9.052(3)	13.357(6)	13.370(10)
*α*,°	90	101.925(12)	114.54(2)	114.34(2)
*β*, °	109.932(11)	100.871(11)	100.74(2)	100.91(2)
*γ*, °	90	114.455(10)	102.03(2)	102.07(2)
*V*, Å^3^	2484.6(14)	1221.3(7)	1225.1(9)	1233.7(15)
*Z*	2	1	1	1
*ρ*_calcd_, g cm^−3^	1.242	1.298	1.294	1.294
*μ*, mm^−1^	0.423	0.528	0.572	0.672
*GOF*	1.024	1.054	1.054	1.055
*R*1^a^	0.0615	0.0571	0.0570	0.0817
*wR*2	0.1978	0.1723	0.1799	0.2533

^a^ *I* > 2*σ*(*I*).

**Table 2:** Selected bond lengths (Å) and angles (°) for **1**.

Co–O(1)	2.094(2)	N(11)–C(13)	1.327(5)
Co–O(11)	2.088(2)	C(13)–N(12)	1.323(5)
Co–O(21)	2.112(2)	N(12)–C(14)	1.456(7)
O(1)–C(3)	1.262(4)	C(14)–C(15)	1.417(8)
O(11)–C(13)	1.267(4)	C(21)–C(22)	1.451(8)
O(21)–C(23)	1.256(4)	C(22)–N(21)	1.456(5)
C(1)–C(2)	1.486(6)	N(21)–C(23)	1.325(5)
C(2)–N(1)	1.450(5)	C(23)–N(22)	1.335(5)
N(1)–C(3)	1.331(5)	N(22)–C(24)	1.450(6)
C(3)–N(2)	1.325(5)	C(24)–C(25)	1.390(9)
N(2)–C(4)	1.462(7)	B–F(1)	1.279(6)
C(4)–C(5)	1.372(9)	B–F(2)	1.311(9)
C(11)–C(12)	1.289(9)	B–F(3)	1.331(8)
C(12)–N(11)	1.459(6)	B–F(4)	1.265(10)

O(11)#1–Co–O(11)	180.00(19)	C(3)–N(2)–C(4)	124.0(4)
O(11)#1–Co–O(1)	92.93(10)	C(5)–C(4)–N(2)	111.7(6)
O(11)–Co–O(1)	87.07(10)	C(11)–C(12)–N(11)	119.0(7)
O(11)#1–Co–O(1)#1	87.07(10)	C(13)–N(11)–C(12)	126.9(4)
O(11)–Co–O(1)#1	92.93(10)	O(11)–C(13)–N(12)	119.6(4)
O(1)–Co–O(1)#1	180.00(8)	O(11)–C(13)–N(11)	120.4(3)
O(11)#1–Co–O(21)#1	86.69(10)	N(12)–C(13)–N(11)	120.0(4)
O(11)–Co–O(21)#1	93.31(10)	C(13)–N(12)–C(14)	125.1(4)
O(1)–Co–O(21)#1	87.20(10)	C(15)–C(14)–N(12)	112.8(6)
O(1)#1–Co–O(21)#1	92.80(10)	C(21)–C(22)–N(21)	113.9(5)
O(11)#1–Co–O(21)	93.31(10)	C(23)–N(21)–C(22)	127.9(4)
O(11)–Co–O(21)	86.69(10)	O(21)–C(23)–N(21)	121.0(3)
O(1)–Co–O(21)	92.80(10)	O(21)–C(23)–N(22)	120.1(3)
O(1)#1–Co–O(21)	87.20(10)	N(21)–C(23)–N(22)	119.0(3)
O(21)#1–Co–O(21)	180.00(12)	C(23)–N(22)–C(24)	124.3(4)
C(3)–O(1)–Co	132.5(2)	C(25)–C(24)–N(22)	114.1(6)
C(13)–O(11)–Co	127.6(2)	F(4)–B–F(1)	111.2(9)
C(23)–O(21)–Co	129.7(2)	F(4)–B–F(2)	103.0(7)
N(1)–C(2)–C(1)	111.0(4)	F(1)–B–F(2)	115.0(6)
C(3)-N(1)-C(2)	125.8(3)	F(4)-B-F(3)	105.1(8)
O(1)-C(3)-N(2)	119.9(4)	F(1)-B-F(3)	115.1(5)
O(1)-C(3)-N(1)	121.2(3)	F(2)-B-F(3)	106.3(7)
N(2)-C(3)-N(1)	118.9(4)		

Symmetry transformation used to generate equivalent atoms: #1 −*x*,−*y*,−*z*.

**Table 3:** Selected bond lengths (Å) and angles (°) for **2**.

Co–O(1)	2.090(2)	N(11)–C(13)	1.329(4)
Co–O(11)	2.100(2)	C(13)–N(12)	1.323(5)
Co–O(21)	2.097(2)	N(12)–C(14)	1.446(5)
O(1)–C(3)	1.259(4)	C(14)–C(15)	1.213(9)
O(11)–C(13)	1.260(4)	C(21)–C(22)	1.424(7)
O(21)–C(23)	1.265(4)	C(22)–N(21)	1.456(6)
C(1)–C(2)	1.329(8)	N(21)–C(23)	1.330(4)
C(2)–N(1)	1.455(6)	C(23)–N(22)	1.324(4)
N(1)–C(3)	1.329(4)	N(22)–C(24)	1.457(5)
C(3)–N(2)	1.328(4)	C(24)–C(25)	1.489(7)
N(2)–C(4)	1.455(4)	Cl–O(34)	1.257(8)
C(4)–C(5)	1.488(6)	Cl–O(32)	1.295(5)
C(11)–C(12)	1.456(7)	Cl–O(31)	1.307(5)
C(12)–N(11)	1.455(6)	Cl–O(33)	1.386(8)

O(1)#1–Co–O(1)	180.00(9)	C(3)–N(2)–C(4)	125.7(3)
O(1)#1–Co–O(21)	86.56(9)	N(2)–C(4)–C(5)	110.8(4)
O(1)–Co–O(21)	93.44(9)	N(11)–C(12)–C(11)	112.4(4)
O(1)#1–Co–O(21)#1	93.44(9)	C(13)–N(11)–C(12)	123.5(3)
O(1)–Co–O(21)#1	86.56(9)	O(11)–C(13)–N(12)	121.2(3)
O(21)–Co–O(21)#1	180.00(16)	O(11)–C(13)–N(11)	120.4(3)
O(1)#1–Co–O(11)	93.24(9)	N(12)–C(13)–N(11)	118.4(3)
O(1)–Co–O(11)	86.76(9)	C(13)–N(12)–C(14)	127.8(4)
O(21)–Co–O(11)	86.22(8)	C(15)–C(14)–N(12)	120.2(5)
O(21)#1–Co–O(11)	93.78(8)	C(21)–C(22)–N(21)	113.4(5)
O(1)#1–Co–O(11)#1	86.76(9)	C(23)–N(21)–C(22)	123.9(3)
O(1)–Co–O(11)#1	93.24(9)	O(21)–C(23)–N(22)	121.1(3)
O(21)–Co–O(11)#1	93.78(8)	O(21)–C(23)–N(21)	120.3(3)
O(21)#1–Co–O(11)#1	86.22(8)	N(22)–C(23)–N(21)	118.6(3)
O(11)–Co–O(11)#1	180.00(11)	C(23)–N(22)–C(24)	126.2(3)
C(3)–O(1)–Co	129.82(19)	N(22)–C(24)–C(25)	110.0(4)
C(13)–O(11)–Co	129.3(2)	O(34)–Cl–O(32)	114.5(8)
C(23)–O(21)–Co	129.46(18)	O(34)–Cl–O(31)	119.6(7)
C(1)–C(2)–N(1)	115.3(6)	O(32)–Cl–O(31)	112.1(5)
C(3)–N(1)–C(2)	123.4(3)	O(34)–Cl–O(33)	98.7(9)
O(1)–C(3)–N(2)	121.6(3)	O(32)–Cl–O(33)	105.0(7)
O(1)–C(3)–N(1)	119.8(3)	O(31)–Cl–O(33)	104.2(6)
N(2)–C(3)–N(1)	118.5(3)		

Symmetry transformation used to generate equivalent atoms: #1 −*x*, −y, −*z*.

**Table 4:** Selected bond lengths (Å) and angles (°) for **3**.

Ni–O(1)	2.068(2)	N(11)–C(13)	1.323(5)
Ni–O(11)	2.073(2)	C(13)–N(12)	1.334(5)
Ni–O(21)	2.076(2)	N(12)–C(14)	1.461(6)
O(1)–C(3)	1.257(4)	C(14)–C(15)	1.415(8)
O(11)–C(13)	1.266(4)	C(21)–C(22)	1.337(16)
O(21)–C(23)	1.263(4)	C(22)–N(21)	1.460(5)
C(1)–C(2)	1.498(7)	N(21)–C(23)	1.322(5)
C(2)–N(1)	1.462(5)	C(23)–N(22)	1.335(5)
N(1)–C(3)	1.329(5)	N(22)–C(24)	1.454(6)
C(3)–N(2)	1.336(5)	C(24)–C(25)	1.462(8)
N(2)–C(4)	1.458(7)	Cl–O(34)	1.274(8)
C(4)–C(5)	1.368(9)	Cl–O(32)	1.298(5)
C(11)–C(12)	1.488(7)	Cl–O(31)	1.310(6)
C(12)–N(11)	1.459(5)	Cl–O(33)	1.378(9)

O(1)#1–Ni–O(1)	180.00(11)	C(3)–N(2)–C(4)	123.0(4)
O(1)#1–Ni–O(11)	86.68(10)	C(5)–C(4)–N(2)	114.0(6)
O(1)–Ni–O(11)	93.32(10)	N(11)–C(12)–C(11)	110.5(4)
O(1)#1–Ni–O(11)#1	93.32(10)	C(13)–N(11)–C(12)	126.5(3)
O(1)–Ni–O(11)#1	86.68(10)	O(11)–C(13)–N(11)	121.6(3)
O(11)–Ni–O(11)#1	180.00(17)	O(11)–C(13)–N(12)	120.0(3)
O(1)#1–Ni–O(21)#1	86.72(10)	N(11)–C(13)–N(12)	118.4(3)
O(1)–Ni–O(21)#1	93.28(10)	C(13)–N(12)–C(14)	123.8(4)
O(11)–Ni–O(21)#1	93.76(10)	C(15)–C(14)-N(12)	113.9(5)
O(11)#1–Ni–O(21)#1	86.24(10)	C(21)–C(22)–N(21)	117.1(9)
O(1)#1–Ni–O(21)	93.28(10)	C(23)–N(21)–C(22)	128.0(4)
O(1)–Ni–O(21)	86.72(10)	O(21)–C(23)–N(21)	121.5(3)
O(11)–Ni–O(21)	86.24(10)	O(21)–C(23)–N(22)	119.7(4)
O(11)#1–Ni–O(21)	93.76(10)	N(21)–C(23)–N(22)	118.8(3)
O(21)#1–Ni–O(21)	180.00(13)	C(23)–N(22)–C(24)	123.8(4)
C(3)–O(1)–Ni	130.2(2)	N(22)–C(24)–C(25)	112.7(5)
C(13)–O(11)–Ni	129.4(2)	O(34)–Cl–O(32)	113.6(8)
C(23)–O(21)–Ni	129.5(2)	O(34)–Cl–O(31)	118.5(8)
N(1)–C(2)–C(1)	110.3(4)	O(32)–Cl–O(31)	113.0(5)
C(3)–N(1)–C(2)	125.5(3)	O(34)–Cl–O(33)	98.6(10)
O(1)–C(3)–N(1)	121.9(3)	O(32)–Cl–O(33)	105.1(7)
O(1)–C(3)–N(2)	119.8(3)	O(31)–Cl–O(33)	105.7(7)
N(1)–C(3)–N(2)	118.3(3)		

Symmetry transformation used to generate equivalent atoms: #1 −*x*, −*y*, −*z*.

**Table 5:** Selected bond lengths (Å) and angles (°) for **4**.

Zn–O(1)	2.108(3)	N(11)–C(13)	1.330(6)
Zn–O(11)	2.107(3)	C(13)–N(12)	1.328(6)
Zn–O(21)	2.111(3)	N(12)–C(14)	1.470(9)
O(1)–C(3)	1.265(5)	C(14)–C(15)	1.313(12)
O(11)–C(13)	1.263(5)	C(21)–C(22)	1.208(14)
O(21)–C(23)	1.279(5)	C(22)–N(21)	1.469(7)
C(1)–C(2)	1.485(10)	N(21)–C(23)	1.315(7)
C(2)–N(1)	1.457(6)	C(23)–N(22)	1.328(6)
N(1)–C(3)	1.331(6)	N(22)–C(24)	1.437(9)
C(3)–N(2)	1.337(6)	C(24)–C(25)	1.496(10)
N(2)–C(4)	1.452(8)	Cl–O(34)	1.29(2)
C(4)–C(5)	1.431(10)	Cl–O(32)	1.318(12)
C(11)–C(12)	1.510(9)	Cl–O(31)	1.402(13)
C(12)–N(11)	1.461(6)	Cl–O(33)	1.440(16)

O(11)#1–Zn–O(11)	180.0(2)	C(3)–N(2)–C(4)	124.0(5)
O(11)#1–Zn–O(1)#1	92.91(13)	C(5)–C(4)–N(2)	113.5(7)
O(11)–Zn–O(1)#1	87.09(13)	N(11)–C(12)–C(11)	109.7(5)
O(11)#1–Zn–O(1)	87.09(13)	C(13)–N(11)–C(12)	124.8(4)
O(11)–Zn–O(1)	92.91(13)	O(11)–C(13)–N(12)	119.8(4)
O(1)#1–Zn–O(1)	180.00	O(11)–C(13)–N(11)	121.5(4)
O(11)#1–Zn–O(21)	92.83(12)	N(12)–C(13)–N(11)	118.7(4)
O(11)–Zn–O(21)	87.17(12)	C(13)–N(12)–C(14)	123.6(5)
O(1)#1–Zn–O(21)	93.04(12)	C(15)–C(14)–N(12)	116.1(8)
O(1)–Zn–O(21)	86.96(12)	C(21)–C(22)–N(21)	121.2(8)
O(11)#1–Zn–O(21)#1	87.17(12)	C(23)–N(21)–C(22)	128.4(5)
O(11)–Zn–O(21)#1	92.83(12)	O(21)–C(23)–N(21)	121.3(4)
O(1)#1–Zn–O(21)#1	86.96(12)	O(21)–C(23)–N(22)	119.2(5)
O(1)–Zn–O(21)#1	93.04(12)	N(21)–C(23)–N(22)	119.4(4)
O(21)–Zn–O(21)#1	180.00(18)	C(23)–N(22)–C(24)	124.9(5)
C(3)–O(1)–Zn	129.5(3)	N(22)–C(24)–C(25)	112.6(7)
C(13)–O(11)–Zn	129.6(3)	O(34)–Cl–O(32)	122.9(10)
C(23)–O(21)–Zn	128.7(3)	O(34)–Cl–O(31)	124.7(11)
N(1)–C(2)–C(1)	109.9(5)	O(32)–Cl–O(31)	109.2(7)
C(3)–N(1)–C(2)	126.0(4)	O(34)–Cl–O(33)	85.0(9)
O(1)–C(3)–N(1)	120.6(4)	O(32)–Cl–O(33)	106.6(8)
O(1)–C(3)–N(2)	120.5(4)	O(31)–Cl–O(33)	97.5(8)
N(1)–C(3)–N(2)	118.9(4)		

Symmetry transformation used to generate equivalent atoms: #1 −*x*, −*y*, −*z*.

**Table 6:** Dimensions of the unique hydrogen bonds (distances in Å and angles in °) for complex **1**.^†^

D^‡^–H⋯A^§^	D^‡^⋯A^§^	H⋯A^§^	<D^‡^HA^§^
N(1)–H(1)⋯O(11)a	2.952(1)	2.177(1)	158.68(3)
N(11)–H(11)⋯O(21)a	2.878(1)	1.983(1)	156.88(2)
N(21)–H(21)⋯O(1)	2.861(1)	2.060(1)	160.05(3)
N(2)–H(2)⋯F(1)b	2.930(1)	2.272(1)	159.06(3)
N(12)–H(12)⋯F(2)c	2.947(1)	2.247(1)	163.27(3)
N(22)–H(22)⋯F(3)	2.964(1)	2.135(1)	151.44(2)

^†^Symmetry transformation used to generate equivalent atoms: a −*x*, −*y*, −*z*; b 1−*x*, 0.5+*y*, 0.5−*z*; c 1−*x*, −*y*, 1−*z*.

^‡^D = donor atom.

^§^A = acceptor atom.

**Table 7:** Dimensions of the unique hydrogen bonds (distances in Å and angles in °) for complex **2**.^†^

D^‡^–H⋯A^§^	D^‡^⋯A^§^	H⋯A^§^	<D^‡^HA^§^
N(2)–H(2)⋯O(21)	2.905(1)	2.154(1)	155.07(4)
N(12)–H(12)⋯O(1)a	2.914(1)	2.177(1)	159.29(3)
N(22)–H(22)⋯O(11)a	2.908(1)	2.153(1)	153.49(2)
N(1)–H(1)⋯O(31)b	3.081(1)	2.381(1)	151.68(3)
N(11)–H(11)⋯O(32)	3.013(1)	2.234(1)	150.97(2)
N(21)–H(21)⋯O(33)c	3.072(1)	2.386(1)	160.41(5)

^†^Symmetry transformation used to generate equivalent atoms: a −*x*, −*y*, −*z*; b −*x*, 1−*y*, 1−*z*; c 1−*x*, 1−*y*, 1−*z*.

^‡^D = donor atom.

^§^A = acceptor atom.

**Table 8:** Dimensions of the unique hydrogen bonds (distances in Å and angles in °) for complex **3**.^†^

D^‡^–H⋯A^§^	D^‡^⋯A^§^	H⋯A^§^	<D^‡^HA^§^
N(1)–H(1)⋯O(11)	2.886(1)	2.055(1)	156.94(5)
N(11)–H(11)⋯O(21)a	2.892(1)	2.082(1)	155.01(5)
N(21)–H(21)⋯O(1)a	2.890(2)	2.091(1)	154.16(6)
N(2)–H(2)⋯O(31)	3.097(1)	2.343(1)	159.73(6)
N(12)–H(12)⋯O(33)b	3.077(2)	2.390(1)	156.88(5)
N(22)–H(22)⋯O(32)c	3.038(1)	2.369(1)	144.26(5)

^†^Symmetry transformation used to generate equivalent atoms: a −*x*, −*y*, −*z*; b *x*, *y*, 1 + *z*; c 1 − *x*, 1 − *y*, −*z*.

^‡^D = donor atom.

^§^A = acceptor atom.

**Table 9:** Dimensions of the unique hydrogen bonds (distances in Å and angles in °) for complex **4**.^†^

D^‡^–H⋯A^§^	D^‡^⋯A^§^	H⋯A^§^	<D^‡^HA^§^
N(1)–H(1)⋯O(21)a	2.905(2)	1.876(1)	150.35(6)
N(11)–H(11)⋯O(1)	2.904(2)	1.932(1)	152.80(6)
N(21)–H(21)⋯O(11)a	2.921(2)	2.314(2)	162.75(10)
N(2)–H(2)⋯O(33)	3.153(2)	2.392(2)	160.19(8)
N(12)–H(12)⋯O(31)b	3.086(2)	2.234(1)	155.95(7)
N(22)–H(22)⋯O(32)c	3.035(2)	2.365(1)	150.81(8)

^†^Symmetry transformation used to generate equivalent atoms: a −*x*, −*y*, −*z*; b *x*, *y*, 1 − *z*; c 1 − *x*, 1 − *y*, 1 − *z*.

^‡^D = donor atom.

^§^A = acceptor atom.

**Table 10:** Most characteristic and diagnostic IR fundamentals (cm^−1^) for DEU and complexes **1**–**4**.^a^

Assignments	DEU	**1**	**2**	**3**	**4**
**ν**(NH)	3342 sb	3332 sb	3332 sb	3328 sb	3340 sb
**ν**(CH)	2973 s, 2932 m,	2976 s, 2934 m,	2972 s, 2934 m,	2976 m, 2934 w,	2972 s, 2932 m,
	2874 m	2878 m	2876 w	2876 w	2876 w
_as_(CN)_amide_ + *δ*_as_ *ν*(NH)	1625 vs	1576 vs	1570 vs	1570 vs	1582 vs
**ν**(CO)	1586 vs	1626 vs	1628 vs	1636 vs	1624 vs
*δ*_s_(NH)	1540 m sh	1454 m	1452 w	1450 m	1456 w
*δ*_as_(NH) + _as_(CN)_amide_ *ν*	1259 m	1338 m	1338 w	1334 w	1334 w

^a^KBr pellets.
